# The Diversity of Sequence and Chromosomal Distribution of New Transposable Element-Related Segments in the Rye Genome Revealed by FISH and Lineage Annotation

**DOI:** 10.3389/fpls.2017.01706

**Published:** 2017-10-04

**Authors:** Yingxin Zhang, Chengming Fan, Shuangshuang Li, Yuhong Chen, Richard R.-C. Wang, Xiangqi Zhang, Fangpu Han, Zanmin Hu

**Affiliations:** ^1^Institute of Genetics and Developmental Biology, Chinese Academy of Sciences, Beijing, China; ^2^Center for Life Science, University of Chinese Academy of Sciences, Beijing, China; ^3^Department of Life Science, Henan Normal University, Xinxiang, China; ^4^Forage and Range Research Laboratory, United States Department of Agriculture, Agricultural Research Service, Utah State University, Logan, UT, United States

**Keywords:** fluoresence *in situ* hybridization, nested transposition, *Secale cereale*, TE lineages, variation

## Abstract

Transposable elements (TEs) in plant genomes exhibit a great variety of structure, sequence content and copy number, making them important drivers for species diversity and genome evolution. Even though a genome-wide statistic summary of TEs in rye has been obtained using high-throughput DNA sequencing technology, the accurate diversity of TEs in rye, as well as their chromosomal distribution and evolution, remains elusive due to the repetitive sequence assembling problems and the high dynamic and nested nature of TEs. In this study, using genomic plasmid library construction combined with dot-blot hybridization and fluorescence *in situ* hybridization (FISH) analysis, we successfully isolated 70 unique FISH-positive TE-related sequences including 47 rye genome specific ones: 30 showed homology or partial homology with previously FISH characterized sequences and 40 have not been characterized. Among the 70 sequences, 48 sequences carried Ty3/*gypsy*-derived segments, 7 sequences carried Ty1/*copia*-derived segments and 15 sequences carried segments homologous with multiple TE families. 26 TE lineages were found in the 70 sequences, and among these lineages, *Wilma* was found in sequences dispersed in all chromosome regions except telomeric positions; *Abiba* was found in sequences predominantly located at pericentromeric and centromeric positions; *Wis, Carmilla*, and *Inga* were found in sequences displaying signals dispersed from distal regions toward pericentromeric positions; except DNA transposon lineages, all the other lineages were found in sequences displaying signals dispersed from proximal regions toward distal regions. A high percentage (21.4%) of chimeric sequences were identified in this study and their high abundance in rye genome suggested that new TEs might form through recombination and nested transposition. Our results also gave proofs that diverse TE lineages were arranged at centromeric and pericentromeric positions in rye, and lineages like *Abiba* might play a role in their structural organization and function. All these results might help in understanding the diversity and evolution of TEs in rye, as well as their driving forces in rye genome organization and evolution.

## Introduction

Transposable elements (TEs) represented a high percentage of eukaryotic genomes, 58.58% in *Pinus taeda* (Wegrzyn et al., 2014), 63% in *Sorghum bicolor* (Paterson et al., 2008), 80% in maize (Feschotte et al., 2002), and more than 72% in *Secale cereale* (Bauer et al., 2017). Besides their high copy number, the serial transposition of individual TEs into previously inserted elements can form large nested structures in genomes (Bergman et al., 2006; Bousios et al., 2016). It was proposed that such clustered, scrambled TE nests could be subsequently copied and amplified, and resulted in large amount of duplications of TE nests in genome (Bergman et al., 2006; Coline et al., 2014), and might even form new TE families (Losada et al., 1999). As a consequence of their variety in structure, size, mechanisms of transposition and high copy number, TEs contribute a lot to the genomic rearrangement, nucleotide diversity and speciation (Middleton et al., 2013; Belyayev, 2014).

Besides the high abundance, structure and sequence diversity of TEs in plant genomes, they were also presented distribution variation among lineages. In *Triticum boeoticum*, for instance, a Ty3/*gypsy* lineage *Wgel* was preferentially clustered at both the centromeric and pericentromeric positions, while another two Ty3/*gypsy* lineages (*Erika* and *Sukkula*) were rare at the centromeric positions (Liu et al., 2008). Even the same TE lineage might show diversity between species and ploidy levels. As proved by four Ty3/*gypsy* lineages (*CRM, Athila, Del*, and *Tat*) in *Brachiaria*, evident differences in location and abundance were observed between diploids and polyploidy (Santos et al., 2015). *CRM* (centromeric retrotransposon in maize) is a special Ty3/gypsy element located at centromeric positions of maize, and CHIP assays demonstrated that this element can interact with CENH3 (centromere-specific H3 histone) throughout its length (Zhong et al., 2002). *CRR* (centromeric retrotransposon in rice) and *CRW* (centromeric retrotransposon in wheat), belonging to the same family with *CRM*, were also proved to interact with CENH3 (Nagaki et al., 2004; Li et al., 2013), which suggesting that the Centromeric Retrotransposon (CR) family in grass species played an important role in centromere structural organization and function (Zhong et al., 2002).

Rye (*Secale cereale* L., 2n = 2x = 14) is an important member of the Triticeae, with a high percentage of repetitive elements of more than 92% (Bartoš et al., 2008). Analysis of repetitive sequences in rye has been performed since the 1970s (Weimarck, 1975; Appels et al., 1978), thereafter, many sequences including some TE derived sequences were located and extensively investigated, such as the *Secale* dispersed repeat sequence R173 elements, a rye-specific family distributed in a dispersed manner over all rye chromosomes (Rogowsky et al., 1992); the *Secale* pSc20H family, which was identified as retrotransposon related sequence, and dispersed throughout the rye genome except telomeric positions and nucleolar organizing regions (Ko et al., 2002; Tang et al., 2011); the transposon-like gene *Revolver*, which is dispersed on all seven chromosomes of rye (Tomita, 2008); the *Superior* families, a transposon-like gene family also dispersed in the rye genome (Tomita et al., 2009); the *Secale cereale* clone B2465 retrotransposon Ty3/*gypsy*-like sequence, which displayed strong hybridization signals on rye chromosomes (Carchilan et al., 2009); the predominantly pericentromere-located pSc10C families (Ko et al., 2002); the centromere-located Ty1-*copia* retrotransposons of the *Bilby* family (Francki, 2001) and the centromere-located Sc192 bp repeats, which were identified as Ty3/*gypsy*-type sequences (Banaei-Moghaddam et al., 2012).

Even though some TEs have been cytologically defined, and great progress has been achieved in rye genome sequencing and expressed sequence tags analysis (Martis et al., 2013; Bauer et al., 2017), there remains a limited understanding about the constitution, chromosomal distribution, diversity and abundance of TEs in rye. In addition, due to the complex organization of TEs and the assembly problem caused by them, the whole genome-wide analysis may not accurately reflect the TE distribution and abundance for any region of the genome (Bergman et al., 2006), especially for genomes haven’t been successfully assembled. The fluorescence *in situ* hybridization (FISH) technique, which was developed by Langer-Safer et al. (1982), was popular for physical mapping of high copy number sequences clustered in plant genomes (Iwata-Otsubo et al., 2016; Gouveia et al., 2017). Thus the FISH technique provided an efficient tool to locate the hardly assembled TE sequences on chromosomes of rye (Li et al., 2016).

To gain more insight into the diversity of sequences and chromosomal distribution of TEs and their evolution in rye, we isolated 70 unique FISH-positive TE-related sequences and investigated their chromosomal location and sequence composition using FISH and TE lineage annotation. 26 TE lineages were found in these newly identified sequences and variable chromosomal distribution bias were observed among these TE lineages; additionally, TE lineage *Abiba* was both found in sequences located at pericentromeric positions and sequences located at centromeric positions. Our results might provide new information for the highly dynamic nature of TEs in rye and their important roles in driving genome diversity, evolution and speciation, as well as centromere organization.

## Materials and Methods

### Plant Materials

The materials used in this work included *Secale cereale* var. King II rye (2n = 2x = 14, R genome), Allohexaploid triticale (AABBRR, 2n = 2x = 42) and *Triticum aestivum* L. var. Chinese Spring wheat (AABBDD, 2n = 2x = 42). To quickly identify rye chromosome specific sequences, allohexaploid triticale (AABBRR, 2n = 2x = 42) was used for the first round of FISH. For sequences displaying signals on A, B and R chromosomes, a second round of FISH was preformed using King II rye and Chinese Spring wheat to check if signals on A, B, and R chromosomes in allohexaploid triticale coincided with those in rye and wheat. The universal probe pSc119.2 was used to help identify chromosomes from rye. The plants used for DNA isolation were grown in the greenhouse with 16 h of lights and 8 h in the dark at 25°C.

### Genomic Plasmid Library Construction

A rye (var. King II) plasmid library for repetitive element screening was constructed by partially digesting the rye genomic DNA using *Hind* III (Takara Bio, Shiga, Japan). The DNA of rye seedlings was extracted using the CTAB method, and the restriction digestion with *Hind III* was performed in a 200 μl reaction with 20 μg genomic DNA, 1× Buffer, sterile H_2_O, and 200 U of *Hind III*. The DNA was digested at 37°C for 20 min and then separated on a 1% agarose gel by electrophoresis. The fraction of 1,000–2,000 bp was collected using an EasyPure Quick Gel extraction kit (TransGen Biotech, Beijing, China). The recovered fragments were ligated into pUC118 vector (Takara Bio, Shiga, Japan) using the TaKaRa DNA ligation kit (Takara Bio, Shiga, Japan) and transformed into competent *E. coli* DH5α (TransGen Biotech, Beijing, China) according to the manufacturer’s instructions.

### Library Screening

Transformed clones were screened using dot-blot hybridization, following the method described by Zhang et al. (2016). For probe labeling, the rye genomic DNA was labeled by digoxigenin-11-dUTP with a random primer DNA labeling kit (Takara Bio, Shiga, Japan) according to the manufacturer’s instructions, but using 1× DIG DNA labeling mix instead of the dNTP in the kit. The darker blots, which were interpreted as high copy number repetitive sequences, were then used in subsequent FISH for chromosomal distribution analysis and sequence identification.

### Slide Preparation and FISH Identification of the Sequences

Slides for FISH were prepared according to Han et al. (2006) and Fu et al. (2015), with minor modifications. Generally, the actively growing root tips were treated with 1.0 MPa nitrous oxide gas (N_2_O) for 2 h, then fixed in 90% glacial acetic acid for 10 min on ice. The root tips could be used immediately or stored in 70% ethanol at -20°C. The root tips were washed three times and digested at 37°C for 1 h in an enzyme solution of 0.5% pectolyase Y-23 (Kikkoman, Co., Tokyo, Japan) and 1% cellulose Onozuka R-10 (Yakult Honsha, Co., Ltd., Minato-ku, Tokyo, Japan) dissolved in citric buffer (10 mM NaC, 10 mM EDTA, pH 5.5). After digestion, the root sections were washed with 70% ethanol and mashed with forceps. The cells were washed with 100% ethanol, resuspended in 100% acetic acid and dropped onto clean glass slides.

For probe labeling, the plasmids carrying subject sequences were labeled with Texas Red-5-dCTP using a nick translation procedure (Han et al., 2006). The labeled probes were dissolved in 2× SSC and 1× TE (20 ng μl^-1^), dropped to the chromosome spreads and denatured together by heating at 100°C for 5 min. Image capturing was carried out using a Nikon Ni-E fluorescence microscope (Nikon, Tokyo, Japan) and further processed with Photoshop 5.0 (Adobe).

### Homology-Based Sequence Identification

The clones were sequenced in both directions with the universal M13 primers synthesized by AuGCT Biotechnology (AuGCT, China) using an ABI PRISM 377 DNA sequencer (Applied Biosystems). Next, the sequences were annotated and classified by a homology search against the RepBase (Bao et al., 2015), TREP database (Wicker et al., 2002) and the REdat_9.0_Poaceae section of the PGSB transposon library (Spannagl et al., 2015) with the default settings. According to the rules proposed by Wicker et al. (2007), nested sequences were annotated segmentally and only homologous regions longer than 80 nucleotides were considered. In order to check if these sequences have been characterized, sequences were further queried against the GenBank database using BLASTN for sequence identity analysis, with a threshold e-value ≤ 10^-5^, and without filtering out low complexity regions. The BLAST results based on the four databases were listed in Supplementary Table S1 and sequences showing homology with TEs were performed a last screening using FISH. To visualize the constitution of each sequence and TE lineages found in these sequences, Venn diagrams and pie charts (**Figure 5** and **Supplementary Figure S1**) were created from the BLAST results listed in Supplementary Table S1. Venn diagrams were created using the online tool Venny 2.1.0^1^ and pie charts were drawn using GraphPad Prism 5.

### Immunofluorescence and FISH Assay

Root tips for immunofluorescence assay were prepared and treated according to Guo et al. (2016). After washing with 1× PBS, the slides were incubated with a rabbit monoclonal anti-CENH3 antibody synthesized by MBL (Nagoya, Japan; 1:250) in 1× TNB [100 mM Tris-HCl, 150 mM NaCl, and 0.5% blocking reagent (w/v)] at 4°C overnight in a wet chamber. The rabbit antibodies were detected using fluorescein isothiocyanate-conjugated goat anti-rabbit antibody (1:1,000; Jackson Immuno Research Labs). Before performing FISH, the slides were dehydrated in 70, 90, and 100% ethanol for 5 min. Images were captured using a Nikon Ni-E fluorescence microscope (Nikon, Tokyo, Japan).

## Results

### Isolation and FISH Characterization of Repetitive DNA Sequences from Rye

**FIGURE 1:**
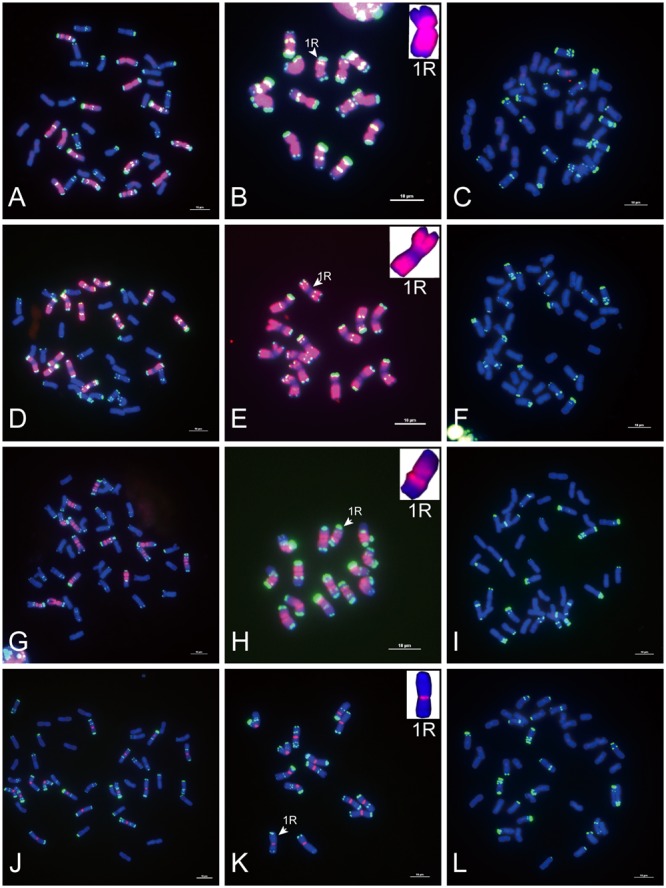
Fluorescence *in situ* hybridization (FISH) mapping of rye species-specific sequences on metaphase chromosomes. Chromosomes were counterstained with DAPI (blue signals), rye species-specific sequences were labeled with Texas Red (red signals), and rye chromosomes were distinguished by pSc 119.2 (green signals). **(A–C)** The signal distribution of HK5-38 on chromosomes of allohexaploid triticale (AABBRR, 2n = 42), *Secale cereale* L. var. King II and “Chinese Spring” wheat (AABBDD, 2n = 42). **(D–F)** The signal distribution of HK11-4 on chromosomes of allohexaploid triticale, *Secale cereale* L. var. King II and “Chinese Spring” wheat. **(G–I)** The signal distribution of HK16-18 on chromosomes of allohexaploid triticale, *Secale cereale* L. var. King II and “Chinese Spring” wheat. **(J–L)** The signal distribution of HK15-13 on chromosomes of allohexaploid triticale, *Secale cereale* L. var. King II and “Chinese Spring” wheat. The signal of each rye species-specific sequence (red signals) was typically displayed by the enlarged 1R chromosomes placed in the inset, with pSc 119.2 (green signals) removed. Bars = 10 μm.

**FIGURE 2:**
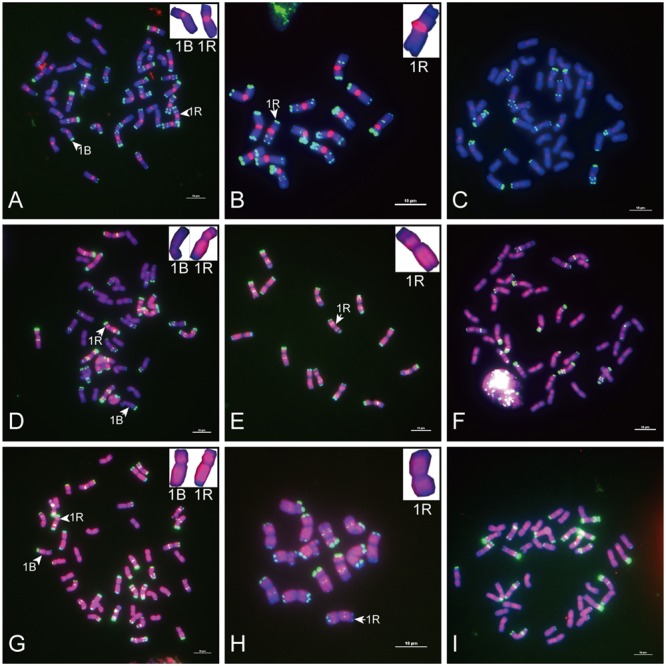
Fluorescence *in situ* hybridization mapping of sequences common to wheat and rye. Metaphase chromosomes were counterstained with DAPI (blue signals), sequences common to wheat and rye were labeled with Texas Red (red signals), and rye chromosomes were distinguished by pSc 119.2 (green signals). **(A–C)** The signal distribution of HK5-64 on chromosomes of allohexaploid triticale, *Secale cereale* L. var. King II and “Chinese Spring” wheat (AABBDD, 2n = 42). **(D–F)** The signal distribution of HK1-68 on chromosomes of allohexaploid triticale, *Secale cereale* L. var. King II and “Chinese Spring” wheat (AABBDD, 2n = 42). **(G–I)** The signal distribution of HK15-21 on chromosomes of allohexaploid triticale, *Secale cereale* L. var. King II and “Chinese Spring” wheat (AABBDD, 2n = 42). The signal of each sequence hybridized with both wheat and rye chromosomes (red signals) was typically displayed by the enlarged 1B and 1R chromosomes placed in the inset, with pSc 119.2 (green signals) removed. Bars = 10 μm.

**FIGURE 3:**
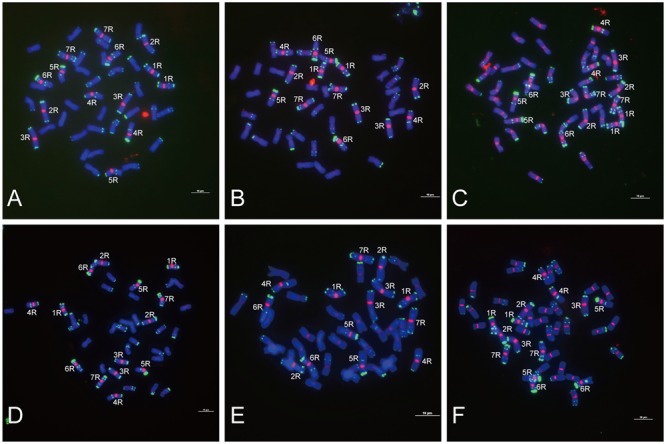
Fluorescence *in situ* hybridization mapping of centromere-specific sequences on allohexaploid triticale metaphase chromosomes. Chromosomes were counterstained with DAPI (blue signals), and rye chromosomes were labeled and distinguished by pSc 119.2 (green signals). **(A)** The signal distribution of HK15-13. **(B)** The signal distribution of HK1-71. **(C)** The signal distribution of HK5-64. **(D)** The signal distribution of HK12-3. **(E)** The signal distribution of HK11-15. **(F)** The signal distribution of HK1-62. Bars = 10 μm.

In this work, a total of 1,800 clones were screened from a *Hind* III-digested rye genomic-DNA library by dot-blot hybridization using rye genomic DNA as the probe. Then, 200 clones appearing as dark dots in the blot hybridization were sequenced and examined for the presence of FISH signals on the metaphase chromosomes of allohexaploid triticale. Furthermore, 70 unique sequences were performed for further analysis after eliminating the 130 duplicate clones or sequences lacking FISH signal. Selected examples are given for all FISH distribution patterns (**Figures 1–3**), and data of all the 70 unique sequences are summarized in **Table 1**.

**Table 1:** Classification and distribution on chromosomes of FISH-positive rye TEs.

			Clone No. and		TE nests (chimeric	Related sequences
			classification		sequences)	
Signal distribution			Retrotransposons			
			Ty3/*gypsy*	Ty1/*copia*		
Part I. Signals enriched in the rye genomes	I-1. Diffused signals on chromosome arms	Group 1. Dispersed from pericentromeric positions to distal regions	HK1-12^c^ HK1-38^c^ HK3-31^c^ HK15-24^c^ HK18-25^c^	HK17-8^c^ HK22-30^c^		
			HK13-27^b^		HK11-47^b^	R173 (Rogowsky et al., 1992)
			HK1-50^b^ HK5-48^b^ HK15-67^b^ HK16-96^b^		HK15-64^b^	*Revolver* (Tomita et al., 2008)
			HK5-78^b^ HK17-84^b^ HK22-67^a^			pSc20H-like sequences (Tang et al., 2011)
		Group 2. Dispersed from distal regions to proximal regions, without obvious signals at pericentromeric positions	HK13-23^c^ HK14-7^c^ HK14-16^c^ HK20-83^c^ HK17-50^c^ HK16-8^c^	HK3-94^c^	HK1-67^c^ HK15-5^c^	
					HK1-56^b^	*Revolver* (Tomita et al., 2008)
			HK17-72^b^			*Secale cereale* clone B2465 (Carchilan et al., 2009)
			HK1-54^b^ HK2-51^b^ HK11-4^a^		HK-52^b^ HK-26^b^ HK11-22^b^ HK17-87^b^	R173 (Rogowsky et al., 1992)
			HK17-82^b^			RXXX630 (Mao et al., 1994)
	I-2. Strong signals at the pericentromeric and centromeric positions	Pericentromeric positions	HK1-36^c^ HK1-59^c^ HK3-21^c^ HK3-93^c^			
			HK3-71^b^ HK18-92^b^		HK16-18^b^	*Superior* (Tomita et al., 2009)
		Centromeric positions	HK11-15^c^			
					HK1-71^b^	Sc192 bp-rye-sortedB-clone-1 (Banaei-Moghaddam et al., 2012)
			HK12-3^b^ HK15-13^a^			*Bilby* (Francki, 2001)
Part II. Signals enriched in both rye and wheat genomes	II-1. Strong signals on the rye chromosomes and less intense signals on wheat chromosomes	Centromeric positions	HK1-62^b^		HK5-64^b^	*Bilby* (Francki, 2001)
		Diffused signals on chromosome arms	HK1-68^c^ HK2-75^c^ HK2-84^c^ HK3-83^c^ HK5-34^c^ HK15-53^c^ HK6-36^c^	HK5-73^c^	HK5-54^c^	
			HK5-83^b^ HK18-68^a^			*Secale cereale* clone B2465 (Carchilan et al., 2009)
	II-2. Strong signals distributed on both rye and wheat chromosomes		HK3-43^c^ HK15-21^c^ HK22-33^c^ HK22-85^c^	HK5-29^c^ HK17-88^c^ HK18-5^c^	HK5-7^c^	
			HK22-24^b^		HK5-70^b^	R173 (Rogowsky et al., 1992)

*Sequences presented here are registered in GenBank as accession numbers KY327841-KY327936. ^a^Sequences showing homology to the reported sequences. ^b^Sequences showing partial homology to the reported sequences. ^c^Newly identified FISH-positive sequences in rye.*

According to the FISH signal patterns, the identified sequences fell into two main categories: signals enriched in the rye genomes (**Table 1**, part I, 47 sequences) and signals enriched in both rye and wheat genomes (**Table 1**, part II, 23 sequences).

**FIGURE 4:**
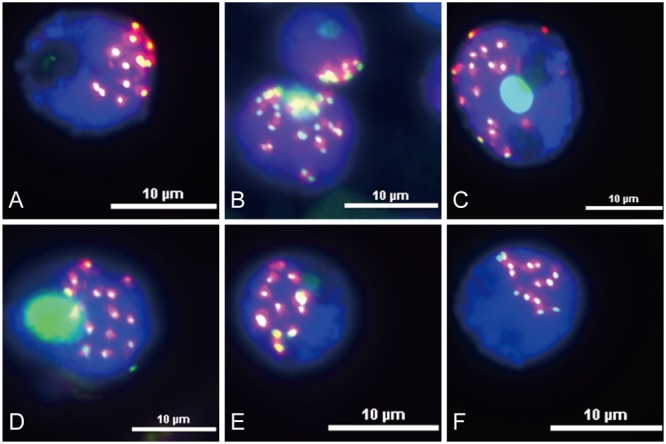
Immuno-colocalization of centromeric sequences and CENH3 at the rye nuclei interphase stage. Nuclei were counterstained with DAPI (blue signals), and CENH3 binding was detected by the secondary antibody anti-rabbit coupled to fluorescein isothiocyanate (FITC; green signals). The centromeric sequences probes were labeled with Texas Red (red signals). **(A)** Co-localization of HK15-13 and CENH3. **(B)** Co-localization of HK1-71 and CENH3. **(C)** Co-localization of HK5-64 and CENH3. **(D)** Co-localization of HK12-3 and CENH. **(E)** Co-localization of HK11-15 and CENH3. **(F)** Co-localization of HK1-62 and CENH3. Bars = 10 μm.

Of the 47 rye genome-specific sequences (**Table 1**, part I), 17 sequences (**Table 1**, I-1, group1) produced signals dispersed from proximal regions toward distal regions of rye chromosomes (**Figures 1A–C**), 19 sequences (**Table 1**, I-1, group2) produced signals dispersed from distal regions to pericentromeric positions of rye chromosomes, without obvious signals at pericentromeric and centromeric positions (**Figures 1D–F**), 7 sequences (**Table 1**, I-2, pericentromeric positions) produced strong signals at pericentromeric positions (**Figures 1G–I**) and 4 sequences (**Table 1**, I-2, centromeric positions) were located at the centromeric regions (**Figures 1J–L, 3A,B,D,E**).

Of the 23 sequences hybridized with both rye and wheat chromosomes (**Table 1**, part II), 13 sequences (**Table 1**, part II-1) displayed stronger signals on rye chromosomes but weaker signals on wheat chromosomes (**Figures 2A–F**), including 2 centromere located sequences (**Figures 2A–C, 3C,F**); 10 sequences (**Table 1**, part II-2) produced same intensely dispersed signals on both rye and wheat chromosomes (**Figures 2G–I**). Among the 23 sequences, only three sequences (HK18-5, HK17-88, and HK5-70) produced signals dispersed from distal regions toward pericentromeric positions, without obvious signals at pericentromeric and centromeric positions. All the other non-centromere located sequences produced signals dispersed from proximal regions toward distal regions (data not shown).

### Immunofluorescence Analysis of Centromere Located Sequences

Functional centromeres are epigenetically specified by incorporation of CENH3, a centromere-specific histone H3 variant (Li et al., 2013; Cech and Peichel, 2016). To determine whether the centromere located sequences are part of the functional areas of centromeres, we conducted immunofluorescence assay and sequential FISH experiments on the same interphase nuclei of King II rye. All the six centromere located sequences were co-localized with CENH3 on all the seven pairs of rye chromosomes (**Figure 4**), but the signals were larger than those of CENH3, which suggested that not all of their sequences were present at the kinetochore positions.

### Annotation of the FISH-Positive Sequences

The FISH-positive fragments were sequenced. All the sequence data were registered in the GenBank as accession numbers (KY327841-KY327936).

**FIGURE 5:**
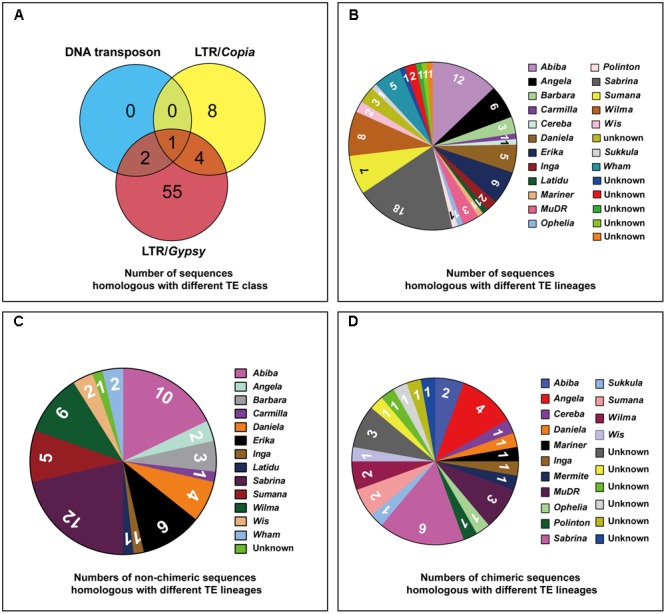
Number of sequences homologous with different TE lineages. **(A)** Venn diagram showing number of sequences homologous with different TE class, the numbers in overlapped regions representing the number of sequences homologous with multiple TE class. **(B)** Pie chart showing numbers of sequences homologous with different TE lineages, six unknown lineages were included. **(C)** Pie chart showing numbers of non-chimeric sequences homologous with different TE lineages, one unknown lineage was included. **(D)** Pie chart showing numbers of chimeric sequences homologous with different TE lineages, six unknown lineage were included.

Based on the homology search, all the 70 isolated sequences were labeled as TE derived sequences: 48 sequences carried Ty3/*gypsy*-derived segments, 7 sequences carried Ty1/*copia*-derived segments and 15 sequences (chimeric sequences) carried segments homologous with multiple TE families (**Table 1**). 26 TE lineages (six unknown lineages were included) were found in these sequences (**Figure 5B** and Supplementary Table S1): 53 sequences carried segments exclusively homologous with TE lineages belonging to Ty3/*gypsy* (seven chimeric sequences included); seven sequences carried segments exclusively homologous with TE lineages belonging to Ty1/*copia* (one chimeric sequences included); four sequences (chimeric sequences) carried segments homologous with TE lineages belonging to Ty3/*gypsy* and Ty1/*copia*; 2 sequences (chimeric sequences) carried segments homologous with TE lineages belonging to Ty3/*gypsy* and DNA transposons; one sequence (chimeric sequence) carried segments homologous with TE lineages belonging to Ty3/*gypsy*, Ty1/*copia* and DNA transposons (**Figure 5A** and Supplementary Table S1). Among these TE lineages (six unknown lineages were not included), four TE lineages were exclusively found in non-chimeric sequences: *Barbara, Carmila, Latidu*, and *Erika*; seven TE lineages were exclusively found in chimeric sequences: *Cereba, Mariner, MuDR, Ophelia, Polinton, Sukkula*, and *Vandal* (*MuDR*); nine TE lineages were found both in non-chimeric and non-chimeric sequences: *Abiba, Angela, Danila, Inga, Wis, Sabrina, Wham, Wilma*, and *Sumana* (**Figure 5** and **Supplementary Figure S1A**, Table S1).

In addition, the frequency of occurrence of different TE lineages in the 70 sequences was also different, such as *Angela* was found in 6 sequences, *Danila* in 5 sequences, *Erika* in 6 sequences, *Inga* in 2 sequences, *Sabrina* in 18 sequences, *Summana* in 7 sequences, *Wilma* in 8 sequences, *Wham* in 5 sequences, *Abiba* in 12 sequences, *Barbara* in 3 sequences, *Wis* in 2 sequences and all the other lineages in only one sequence (**Figure 5B** and Supplementary Table S1). Even though some TE lineages existed in only one sequence, they still presented high copy numbers in the rye genome, inferred from the strong FISH signals displayed by their residing sequences.

As suggested by the FISH patterns each sequence displayed and the TE lineages found in these sequences, differential chromosomal distribution of these TE lineages was detected (**Supplementary Figure S1B** and Table S1): *Abiba* (*Gypsy* type) were found in sequences located at the pericentromeric and centromeric positions; *Cereba* (*Gypsy* type), *Mariner* (DNA transposons) and *MuDR* (DNA transposons) were only found in centromere located sequences; *Latidu* (*Gypsy* type) was only found in HK5-34, which displayed stronger signals on chromosome arms of rye, but weaker on those of wheat; *Vandal* (DNA transposons) was only found in the pericentromere located sequence HK16-18; *Erika* (*Gypsy* type), *Sukkula* (*Gypsy* type), *Carmila* (*Gypsy* type), *Inga* (*Copia* type), *Ophelia* (*Gypsy* type), and *Polinton* (DNA transposons) were all only found in sequences displaying signals on chromosome arms of rye; *Wilma* (*Gypsy* type) was found in sequences dispersed in all chromosome regions except telomeric positions; *Barbara* (*Copia* type) was found in sequences displaying signals on chromosome arms of rye and sequences displaying same intense signals on chromosome arms of both rye and wheat; *Angela* (*Copia* type), *Sabrina* (*Gypsy* type), *Danila* (*Gypsy* type), *Wham* (*Gypsy* type), and *Summana* (*Gypsy* type) were found in sequences dispersed in all chromosome regions except centromeric and telomeric positions, but the last three lineages (*Danila, Wham*, and *Summana*) were not found in sequences producing same intense signals on chromosome arms of both rye and wheat; *Wis* (*Copia* type) was only found in sequences displaying same intense signals on chromosome arms of both rye and wheat. Besides, *Erika, Summana, Barbara, Sukkula*, and *Latidu* (highlighted in green) were only found in sequences displaying signals dispersed from proximal regions toward distal regions; while *Wilma, Danila, Sabrina, Wham*, and *Angela* (highlighted in red) were found in sequences displaying signals dispersed from proximal regions toward distal regions and sequences displaying signals dispersed from distal regions toward pericentromeric positions, without obvious signals at centromeric and pericentromeric positions.

In order to check if these sequences have been cytologically defined, all the sequences were compared with the published repetitive sequences that have been characterized by FISH. The searching results showed that 30 sequences carried fragments homologous with previously FISH identified sequences (**Table 1** and Supplementary Table S1): 4 complete sequences (**Table 1**, sequence names labeled with ‘a’ in the right upper corners) and partial segments of 26 sequences (**Table 1**, sequence names labeled with ‘b’ in the right upper corners). Roughly, 11 sequences showed homology or partial homology with the *Secale* dispersed repeat sequence R173 family (Rogowsky et al., 1992): HK11-4, HK1-54, HK-26, HK22-24, HK5-70, HK13-27, HK-52, HK2-51, HK11-22, and HK17-87; 5 sequences showed partial homology with the *Secale Revolver* transposon sequences (Tomita et al., 2008): HK15-67, HK16-96, HK5-48, HK1-50, and HK15-64; 3 sequences showed partial homology with the *Secale cereale* clone B2465 retrotransposon Ty3/*gypsy*-like sequence (Carchilan et al., 2009): HK17-72, HK5-83, and HK18-68; 3 sequences showed partial homology with the *Secale* pSc20H family (Tang et al., 2011): HK17-84, HK5-78, and HK22-67; 2 sequences showed partial homology with the rye species-specific DNA element *Superior* (Tomita et al., 2009): HK18-92 and HK16-18; 4 sequences showed partial homology with the *Bilby* family (Francki, 2001): HK1-62, HK12-3, HK5-64, and HK15-13; HK1-71 showed partial homology with the *Secale cereale* clone Sc192 bp-rye-sortedB-clone-1 centromere sequence (Banaei-Moghaddam et al., 2012); HK11-47 contained both R173 and B2465-like segments. In addition, repetitive sequences used as FISH markers were also found in some sequences: 3 sequences (HK17-72, HK11-47, and HK18-68) carried segments homologous with *Secale* pSc119.1-like repeat sequence (Mcintyre et al., 1990); 2 sequences (HK-26 and HK17-82) carried segments homologous with RXXX630, a repetitive DNA sequence common to both rye and wheat (Mao et al., 1994). The remaining 40 sequences did not shared any homology with the previously FISH identified sequences, and had not been characterized yet (**Table 1**, sequence names labeled with ‘c’ in the right upper corners); however, the homologs of 38 of them could be found in the *Secale* databases and/or other databases in the GenBank, with similarity ranging from 70 to 100%.

In order to check the relationship between previously FISH defined families and TE lineages, previously FISH identified sequences (R173, *Revolver, Secale cereale* clone B2465, pSc20H, *Bilby, Superior* and *Secale cereale* clone Sc192 bp-rye-sortedB-clone-1) were download and blasted against the same databases as our sequences. It turned out that pSc20H showed full length homology with the *Erika* lineage, B2465 showed full length homology with the *Daniela* lineage, *Superior* and *Bilby* showed partial homology with the *Abiba* lineage, both R173 and *Revolver* families showed homology with multiple TE lineages (Supplementary Table S2).

## Discussion

Because TEs contributed a major part of the Triticeae genomes, understanding their sequence diversity and distribution dynamics can help investigate genome evolution and speciation (Middleton et al., 2013; Bauer et al., 2017). In rye, it is still challenging due to the unfinished whole genome assembly. However, because of their high abundance and chromosomal clustering nature, TEs can be relative easily located on chromosomes using cytological method like FISH (Rogowsky et al., 1992; Francki, 2001; Kalendar et al., 2004; Tomita, 2008, 2010; Carchilan et al., 2009; Tang et al., 2011), especially high copy number TE lineages. In this study, the chromosomal distribution and sequence diversity of 70 TE related sequences were investigated, which would help understand the organization and evolution of TEs in the rye genome.

Transposable elements constitute at least 72% of the rye genome, with 60% LTR retrotransposons and 7% DNA transposons (Bauer et al., 2017). Ty3/*gypsy* and Ty1/*copia* are two major groups of LTR retrotransposons, and Ty3/*gypsy* elements are generally more presented than Ty1/*copia* ones in angiosperms (Dereeper et al., 2013; Natali et al., 2015; Guyot et al., 2016). Besides different abundance, Ty3/*gypsy* are presented more diversity than Ty1/*copia* in plants (Santos et al., 2015). In this study, 62 of the 70 identified sequences contained Ty3/*gypsy*-derived segments, almost five times of those contained Ty1/*copia*-derived sequences (**Figure 5A**). DNA transposons were also found in the identified sequences: *Polinton* in HK-26 (dispersed in interstitial regions of rye chromosomes), *Vandal* (*MuDR*) in HK16-18 (mainly located at the pericentromeric positions), *Mariner* and two *MuDR* in HK5-64 (located at centromeric positions). Our results showed that Ty3/*gypsy* constituted a major part of TEs in rye and DNA transposons lineages might also play a role in centromere structural organization and function.

Complex or hybrid TEs are commonly seen in genomic sequences, these elements might arise from the nested TE integration, intrachromosomal recombination or variant replication (Wicker et al., 2007; Vitte et al., 2013; Gao et al., 2015). These kind of hybrid TE were often clustered in plant genomes, and can spread over distances as large as 200 kb (Choulet et al., 2010). In this study, 15 chimeric sequences (more than 21.4%) were characterized (Supplementary Table S1), which involved nearly all the TE lineages found in this work (**Supplementary Figure S1A** and Table S1). None of these chimeric sequences were head-tail/head TE junction structures, so these sequences should be independent fragments. To further confirm their existence, all the 15 chimeric sequences were searched against the available *Secale* BAC clones deposited in NCBI database and WGS sequence contigs deposited in IPK Rye BLAST Server^2^. Due to the unfinished whole-genome assembly, only two sequences showed full-length homologous with published data: full-length of HK5-7 was found in 5 WGS sequence contigs (e-value = 0), and full length of HK15-5 was found in *Secale* BAC clone (*Secale cereale* BAC956-D7, e-value = 0). The results suggested that these chimeric sequences should exist in rye genome, and might be formed by a series of nested transposition and/or recombination of TEs. Additionally, the strong FISH signals given by those chimeric sequences suggested that they were highly abundant and stretched long distances in the rye genome, which might result from duplication of these nested copies following the nested insertions and recombination, as suggested by Bergman et al. (2006).

Duplication of nested TEs is not a rare phenomenon in eukaryote genomes, which have been widely observed in *Drosophila* (O’hare et al., 2002; Bergman et al., 2006), as well as barley (Wicker et al., 2005) and *Arabidopsis* (Lippman et al., 2004). Moreover, chimeric TEs (TE nests) were mostly found in rye chromosome specific sequences or sequences displaying stronger signals on rye chromosomes than on wheat chromosomes (**Table 1** and Supplementary Table S1). Another example is the well-studied rye genome specific transposon-like gene *Revolver*, which also shows homology with multiple TE lineages (Supplementary Table S2). All these results indicated that nested transposition, recombination among TE lineages and duplication of nested sequences were important driving forces of speciation and genome evolution, and might also be an important mechanism of new TE family formation (Losada et al., 1999).

Transposable elements are relatively neutral elements within genome (Petrov, 2001), which facilitate them accumulating more changes in the genome (Warren et al., 2015). FISH signal intensity could help to evaluate the homology between probes and target genome, as well as the copy numbers of target sequences in genome. In this study, differential FISH signal intensity was observed among TE lineages (**Supplementary Figure S1**, Table S1, and **Table 1**), such as *Wis* and *Barbara* were found in sequences displaying strong signals on wheat chromosomes; while *Carmilla, Inga, Erika*, and *Sukkula* were found in sequences displaying no signals on wheat chromosomes. Besides differences among TE lineages, differential FISH signal intensity was also observed among different members of a same family: *Angela* was found in sequences displaying strong signals, weaker signals and no signals on wheat chromosomes, as well as *Sabrina* and *Wilma*; *Sumana* was found in sequences displaying weaker signals and no signals on wheat chromosomes, as well as *Daniela* and *wham*. These high diversity among TE lineages and different members of a same TE lineages indicated variably evolutionary rate and direction of these TE lineages, which contributed a lot to the genome diversity and speciation.

Transposable elements constitute a considerable proportion of the centromeric DNA sequences in cereals, for instance, 96% of the centromeric DNA of the hexaploid wheat chromosome 3B was TE sequences (Li et al., 2013). Even though the function of centromeres are conserved, TEs located at centromeric positions keep evolving (Ma et al., 2007; Neumann et al., 2011). During species evolution, new TE lineages might form and play a role in centromere structural organization and function, and some ancestor elements might lose their function or head to extinction. In wheat, for instance, satellite repeats lost their ability to bind with CENH3, and might have been replaced by the *CRW* and *Quinta* elements at the functional centromere (Li et al., 2013). In addition, some species might evolve their own species-specific elements, such as *Bilby* family in rye, which are significantly enriched at the centromeric positions of rye chromosomes (Francki, 2001). In this study, four of the six centromere located sequences contained segments homologous with *Bilby* family (**Table 1** and Supplementary Table S1). Except HK1-71, all the centromere located sequences, including *Bilby* family, contained segments homologous with the *Abiba* TE lineage. These results support the idea that retrotransposon families located at centromeric positions in cereals probably derived from a single conventional Ty3/*gypsy* family or a non-autonomous derivative (Langdon et al., 2000; Nagaki et al., 2003), and from an evolutionary perspective, elder families kept being replaced by new emerged families. Immuno-colocalization of the six centromere located sequences with CENH3 suggested that they might involve in the centromere structural organization. To confirm this, more work needs to be performed.

At the centromeric and pericentromeric positions, meiotic recombination is almost completely suppressed (Gore et al., 2009), but rearrangements caused by retrotransposons were frequently detected (Henikoff et al., 2001; Hall et al., 2004; Liu et al., 2008; Li et al., 2013; Wolfgruber et al., 2016). In our study, a chimeric sequence HK5-64 contains segments from Ty3/*gypsy* (*Abiba*), Ty1/*copia* (*Copia3*) and two type of DNA transposon lineages (*MuDR* and *Mariner*), which suggested that recombination events have occurred during the evolution of rye genome. After BLASTed against the NCBI database, only 289 bp length of segment homologous with *Abiba* was found in the Triticum database, which indicated that this chimeric sequence should form after rye and wheat diverged from a common ancestor. However, we failed to obtain its full length, even though a 782 bp length of its segment was found in the released database of rye (Bauer et al., 2017).

Plant pericentromeres were regions physically separating the centromere core from the gene-rich chromosome arms, which were characterized by large TE islands (Sigman and Slotkin, 2016). In this study, the TE lineage *Abiba* was not only found in almost all the centromere located sequences (except HK1-71), but also in all the pericentromere located sequences, which supported the idea that there was similarity between centromeric and perientromeric regions (Gent et al., 2011). However, more TE lineages dispersed in interstitial regions were found at pericentromerc positions, such as *Angela, Barbara, Danila, Erika, Sumana, Latidu, Sabrina*, and *Wham*. This result suggested that pericentromerc regions might share more TE lineages with interstitial regions.

## Conclusion

The rye genome contained a substantial fraction of repetitive sequences, especially TE sequences. Although broad-scale patterns of TE abundance has been investigated in rye using high-throughput DNA sequencing technology (Bartoš et al., 2008; Fluch et al., 2012; Bauer et al., 2017), the accurate diversity of sequence and chromosomal distribution of TEs in rye remains enigmatic due to their dynamic nature and nested transposition. In this work, the constitution and chromosomal distribution of 70 unique FISH-positive TE-related sequences were identified and characterized. Of the 70 sequences, 30 contained segments homologous with previously FISH characterized TE-related sequences and 40 have not been characterized. 62 of the 70 sequences contained Ty3/gypsy-derived sequences (14 chimeric sequences included), which suggested a high percentage of Ty3/gypsy type TEs in rye genome. 26 TE lineages were found in these identified sequences, and almost all of them could be found in chimeric sequences, which suggested wide nested transposition and recombination have happened among these TE lineages in rye genome. In addition, the strong FISH signals produced by the chimeric sequences indicated that TE nested insertions, recombination, and duplication of nested sequences contributed a lot to new TE family formation, rye genome organization and evolution. Except the conserved centromeric retrotransposon *Cereba*, another TE lineage *Abiba* and 3 DNA transposons were also found in centromere located sequences, which suggested that diverse TE lineages were involved in the centromere structural organization in rye. To wholly understand the structure, organization, potential function and transposition mechanisms of our identified TEs, it is necessary to obtain their full lengths in further work. Our studies provided valuable insights into the constitution, distribution and diversity of TEs in the rye genome, which is helpful in understanding the roles of TEs in driving rye genome organization and evolution.

## Author Contributions

YZ, CF, and ZH designed the experiments. YZ conducted the study, processed the data and wrote the manuscript. CF, SL, YC, RW, XZ, FH, and ZH discussed the results and modified the manuscript. All authors have read and approved the final manuscript.

## Conflict of Interest Statement

The authors declare that the research was conducted in the absence of any commercial or financial relationships that could be construed as a potential conflict of interest.

## Footnotes

1 http://bioinfogp.cnb.csic.es/tools/venny/index.html

2 http://webblast.ipk-gatersleben.de/ryeselect/

## Supplementary Material

FIGURE S1 Venn diagram showing TE lineages found in the 70 identified sequences. **(A)** TE lineages were classified based on the types of their residing sequences: non-chimeric sequences or chimeric sequences, the lineages falling in overlapped regions were found in both types of sequences. **(B)** TE lineages were classified based on the FISH patterns displayed by their residing sequences, TE lineages highlighted in green were exclusively found in sequences displaying signals dispersed from proximal regions toward distal regions; TE lineages highlighted in red were found both in sequences displaying signals dispersed from proximal regions toward distal regions and sequences displaying signals dispersed from distal regions toward pericentromeric positions.
