# Corrigendum: The Diversity of Sequence and Chromosomal Distribution of New Transposable Element-Related Segments in the Rye Genome Revealed by FISH and Lineage Annotation

**DOI:** 10.3389/fpls.2017.02020

**Published:** 2017-11-21

**Authors:** Yingxin Zhang, Chengming Fan, Shuangshuang Li, Yuhong Chen, Richard R.-C. Wang, Xiangqi Zhang, Fangpu Han, Zanmin Hu

**Affiliations:** ^1^Institute of Genetics and Developmental Biology, Chinese Academy of Sciences, Beijing, China; ^2^Center for Life Science, University of Chinese Academy of Sciences, Beijing, China; ^3^Department of Life Science, Henan Normal University, Xinxiang, China; ^4^Forage and Range Research Laboratory, United States Department of Agriculture, Agricultural Research Service, Utah State University, Logan, UT, United States

**Keywords:** fluorescence *in situ* hybridization, nested transposition, *Secale cereale*, TE lineages, variation

In the original article, there were typographical errors in the “Materials and Methods” under the “Plant Materials” section.

The correct version of both instances of “(AABBRR, 2n = 2x = 42)” should be “(AABBRR, 2n = 6x = 42)”.

Also, the correct version of “(AABBDD, 2n = 2x = 42)” should be “(AABBDD, 2n = 6x = 42)”.

The authors apologize for these errors and state that this does not change the scientific conclusions of the article in any way.

## Conflict of interest statement

The authors declare that the research was conducted in the absence of any commercial or financial relationships that could be construed as a potential conflict of interest.

