# Supplementary figures and images for: The Diversity of Sequence and Chromosomal Distribution of New Transposable Element-Related Segments in the Rye Genome Revealed by FISH and Lineage Annotation
Source: Front Plant Sci. 2017 Oct 4;8:1706. doi: 10.3389/fpls.2017.01706 (PMC5632726; doi:10.3389/fpls.2017.01706)

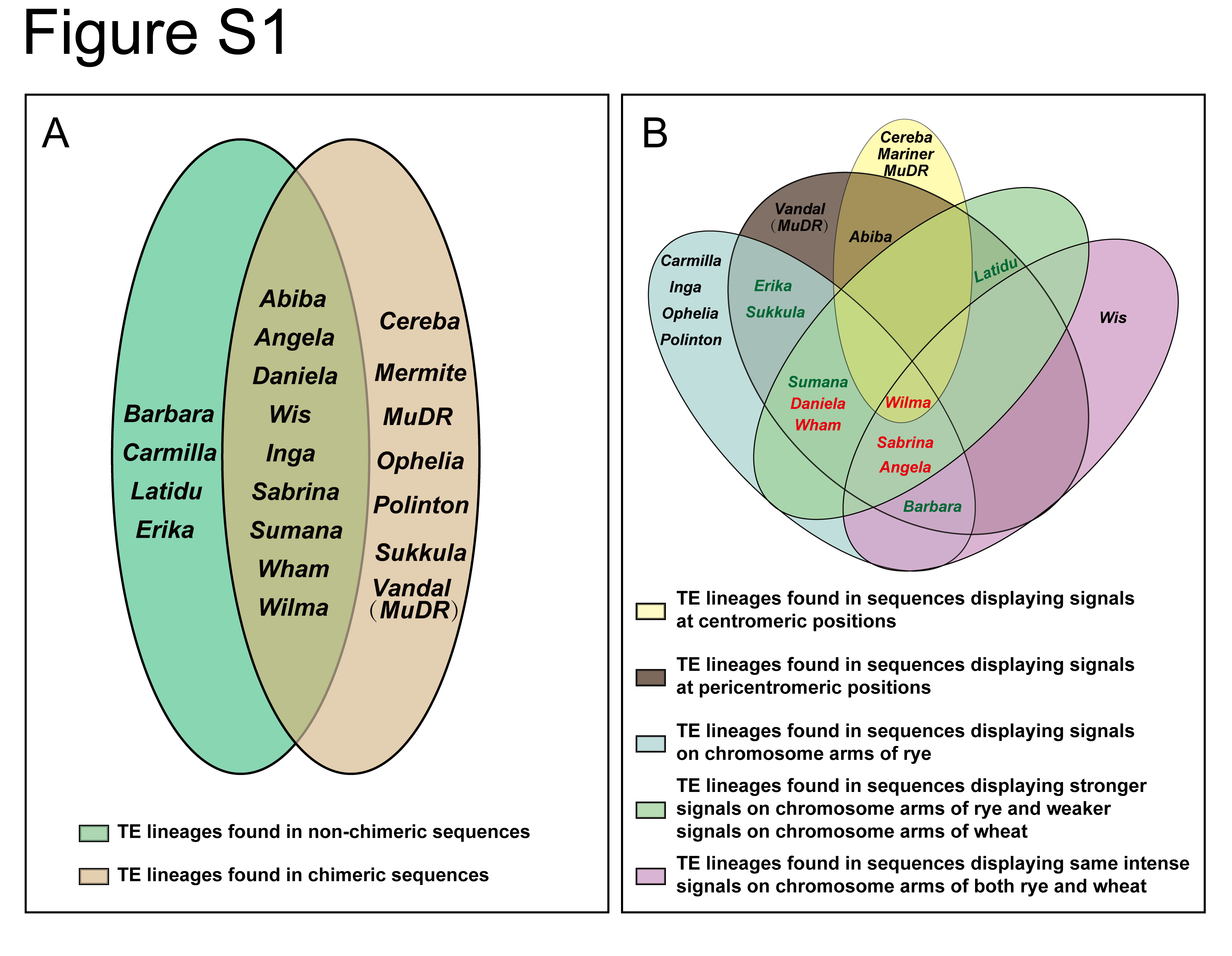

Supplement: FIGURE S1 — Venn diagram showing TE lineages found in the 70 identified sequences. (A) TE lineages were classified based on the types of their residing sequences: non-chimeric sequences or chimeric sequences, the lineages falling in overlapped regions were found in both types of sequences. (B) TE lineages were classified based on the FISH patterns displayed by their residing sequences, TE lineages highlighted in green were exclusively found in sequences displaying signals dispersed from proximal regions toward distal regions; TE lineages highlighted in red were found both in sequences displaying signals dispersed from proximal regions toward distal regions and sequences displaying signals dispersed from distal regions toward pericentromeric positions. [file Image_1.TIF]
